# Glass Polyalkenoate Cements Designed for Cranioplasty Applications: An Evaluation of Their Physical and Mechanical Properties

**DOI:** 10.3390/jfb7020008

**Published:** 2016-03-25

**Authors:** Basel A. Khader, Declan J. Curran, Sean Peel, Mark R. Towler

**Affiliations:** 1 Department of Mechanical & Industrial Engineering, Ryerson University, Toronto, ON M5B 2K3, Canada; basel.khader@ryerson.ca (B.A.K.); curran@ryerson.ca (D.J.C.); 2 Division of Oral & Maxillofacial Surgery & Anaesthesia, Faculty of Dentistry, University of Toronto, Toronto, ON M5G 1G6, Canada; Sean.Peel@dentistry.utoronto.ca

**Keywords:** cranioplasty fixation, germanium, glass polyalkenoate cemets, titanium miniplate, compressive strength, biaxial flexural strength, tensile strength, ovis aries bone

## Abstract

Glass polyalkenoate cements (GPCs) have potential for skeletal cementation. Unfortunately, commercial GPCs all contain, and subsequently release, aluminum ions, which have been implicated in degenerative brain disease. The purpose of this research was to create a series of aluminum-free GPCs constructed from silicate (SiO_2_), calcium (CaO), zinc (ZnO) and sodium (Na_2_O)-containing glasses mixed with poly-acrylic acid (PAA) and to evaluate the potential of these cements for cranioplasty applications. Three glasses were formulated based on the SiO_2_-CaO-ZnO-Na_2_O parent glass (KBT01) with 0.03 mol % (KBT02) and 0.06 mol % (KBT03) germanium (GeO_2_) substituted for ZnO. Each glass was then mixed with 50 wt % of a patented SiO_2_-CaO-ZnO-strontium (SrO) glass composition and the resultant mixtures were subsequently reacted with aqueous PAA (50 wt % addition) to produce three GPCs. The incorporation of Ge in the glass phase was found to result in decreased working (142 s to 112 s) and setting (807 s to 448 s) times for the cements manufactured from them, likely due to the increase in crosslink formation between the Ge-containing glasses and the PAA. Compressive (σ_c_) and biaxial flexural (σ_f_) strengths of the cements were examined at 1, 7 and 30 days post mixing and were found to increase with both maturation and Ge content. The bonding strength of a titanium cylinder (Ti) attached to bone by the cements increased from 0.2 MPa, when placed, to 0.6 MPa, after 14 days maturation. The results of this research indicate that Germano-Silicate based GPCs have suitable handling and mechanical properties for cranioplasty fixation.

## 1. Introduction

Titanium miniplates and screws are used to repair defects in cranioplasty surgeries [1,2]. While these materials can be secured to the surrounding bone using calcium phosphate cements, success has been limited due to the characteristic brittleness of such materials [3,4], meaning that they are unable to resist the forces applied during installation [5]. Poly-methylmethacrylate (PMMA) is often used in cranioplasty but also has limited success; it can cause an impairment of the body’s natural defenses, leaving patients susceptible to infection [6,7]. Almost 7% of PMMA alloplastic cranioplasties suffer some form of displacement and/or fracture [7,8,9,10,11,12,13,14,15,16,17,18]. Additionally, PMMA has a high curing exotherm of up to 120 °C, which can result in necrosis of healthy bone tissue [6], leading to the loosening of the prosthesis [19]; a problem exacerbated by PMMA’s lack of chemical adhesion to bone [20].

Although they have not been used clinically in cranioplasty, Glass Polyalkenoate Cements (GPCs) have potential in this space as, unlike PMMA, they chemically bond with bone. Though the use of GPCs has been restricted to dental [21] and ear, nose and throat (ENT) applications [22,23], attempts have been made to tailor these materials as orthopedic adhesives. For example, titanium has been substituted for silicon in the glass phase of GPCs in order to improve the cement’s mechanical and biological properties [24]. Zinc and silver ions have been added to the glass phase of GPCs because of their antimicrobial activity [25], and strontium ions (Sr^2+^) have been substituted for calcium ions (Ca^2+^) in order to increase radiopacity of the cement and stimulate bone formation around the implantation site [26,27,28]. Germanium (Ge) adopts the role of a network former when incorporated into an ionomer glass, and is theoretically capable of isomorphically replacing Si in the network [29]. Germanium based glasses have previously been investigated by Dickey *et al.* [30,31,32] who reported that glass reactivity decreased with Ge incorporation, resulting in GPCs with extended working times of up to 10 min, setting times between 14 and 36 min, and compression strengths in surplus of 30 MPa after 30 days maturation [30]. Ge was also incorporated into borate-based ionomer glasses (BGG) by Zhang *et al.* [32,33] who reported that its presence increased handling properties of the resultant GPCS formulated from them [32]. This paper expands partly on Dickey and Zhang’s work by investigating the applicability of germano-silicate glasses as components in GPCs for cranioplasty applications. We also build upon the authors own previous work on zinc silicate-based GPCs by evaluating the effect on the physical and mechanical properties of these GPCs by incrementally replacing the Zn in the glass component with Ge [6,34].

## 2. Materials and Methods

### 2.1. Glass Synthesis

Three glass compositions, KBT01, KBT02 and KBT03, were formulated. The control, KBT01, was a SiO_2_-CaO-ZnO-Na_2_O glass; KBT02 and KBT03 contain incremental concentrations of Ge added at the expense of Zn (Table 1). Glasses were prepared by weighing out appropriate amounts of analytical grade reagents and ball milling (1 h). Each mix was then oven dried (100 °C, 1 h) and fired (1500 °C, 1 h) in a pure Silica ceramic crucible and shock quenched into water. The resulting frit was dried, ground and sieved to retrieve a glass powder with a maximum particle size of <45 μm.

A patented Sr-CaO-ZnO-SiO2 (BT101) glass (Table 2) [13,34] was mixed with each KBT glass for the fabrication of the GPC at a 50:50 ratio.

#### Polyacrylic Acids (PAA)

Advanced Healthcare Limited (Kent, UK) supplied the PAA (*M*_w_, 213,000). The acid was freeze-dried and ground and sieved to contain a maximum particle size of <45 μm.

### 2.2. Glass Characterization

#### 2.2.1. Network Connectivity (NC)

The network connectivity of the glasses was calculated using Equation (1) [6] considering that SiO_2_ and GeO_2_ acted as network formers and CaO, ZnO and Na_2_O as network modifiers.

(1) $$\text{NC}=\frac{\text{No}:\text{ BOs}-\text{ No}:\text{ NBOs}}{\text{Total No}:\text{ Bridging species}}$$

where NC = Network Connectivity, BO = Bridging Oxygens, NBO = Non-Bridging Oxygens.

#### 2.2.2. Powder X-ray Diffraction (XRD)

XRD patterns were collected using a PANanlytical X’Pert PRO (PANanlytical Inc., St Laurent, QC, Canada). Glass powder samples were attached to a stainless steel disc using a 20 mm glass slide. The powder compacts were then placed in the X-ray Diffractometer and scanned in the range 10° < 2θ < 80°, at scan step size 0.05° and step time of 10 s. A generator voltage of 45 kV and a tube current of 40 mA were employed using Cu kα X-ray source.

#### 2.2.3. Particle Size Analysis (PSA)

Particle size analysis was performed using a Coulter Ls 100 Fluid module Particle size analyzer (Beckman Coulter, Fullerton, CA, USA). The glass powder samples were evaluated in the range of 0–950 μm with a run length of 60 s. The suspension fluid used in this case was glycerol and was used at a temperature of 37 °C. The relevant volume statistics were calculated on each glass.

### 2.3. Cement Characterization

#### 2.3.1. Cement Preparation

Cements were prepared by first mixing each of the KBT series of glasses (<45 μm; un-annealed) with BT101 glass (un-annealed) at a 1:1 ratio and then combining that mixture with PAA and distilled water on a glass plate at a powder to liquid (P:L) ratio of 1:1 using 1 g of glass powder (0.5 g of KBT and 0.5 g BT101) and a 50 wt % solution of PAA (0.5 g of acid and 0.5 mL of water). The same nomenclature (*i.e.*, KBT01, KBT02 and KBT03) were used for the cements as for the primary glass composition that they were formulated from.

#### 2.3.2. Handling Characteristics (*T*_w_ and *T*_s_)

The cement working time (*T*_w_), measured in ambient temperatures using a stopwatch, was defined as the period of time from the start of mixing during which it was possible to manipulate the material without having an adverse effect on its properties [35].

The setting time of the cements was measured in accordance with ISO9917 [36]. An empty mould was placed on aluminum foil and filled to a level surface with the freshly mixed cement. Sixty seconds after mixing the entire assembly was placed on a metal block (8 mm × 75 mm × 100 mm) in an oven maintained at 37 °C. Ninety seconds after mixing, a Vicat needle indenter (mass, 400 g) was lowered onto the surface of the cement. The needle was allowed to remain on the surface for five seconds, the indent was then observed and the process repeated every thirty seconds until the needle failed to make a complete circular indent when viewed at ×2 magnification. The net setting time of three tests was recorded.

### 2.4. Scanning Electron Microscopy and Energy Dispersive X-ray Analysis (SEM-EDX)

Backscattered electron (BSE) imaging was carried out with a JEOL Co. JSM-6380LV (JEOL Ltd., Tokyo, Japan). Compositional analysis was performed with an EDX Genesis Energy-Dispersive Spectrometer. All EDX spectra were collected at 20 kV using a beam current of 26 nA. Quantitative EDX spectra were subsequently converted into relative concentration data.

### 2.5. Mechanical Properties

#### 2.5.1. Compressive Strength

The compressive strengths (σ_c_) of five cement samples of each formulation were evaluated in ambient air (23 ± 1 °C) according to ISO9917 [36]. Samples were tested after 1, 7 and 30 days using an Instron Universal Testing Systems (Instron Corp, Norwood, MA, USA) fitted with a ±2 kN load cell at a crosshead speed of 1 mm·min^−1^. The moulds, 4 mm Ø, by 6 mm height in line with ISO9917 [36], were filled to excess with freshly mixed cement then covered with acetate sheet. The mould/cement/acetate constructs were then sandwiched between two stainless steel plates, clamped and incubated (37 °C, 1 h). The constructs were subsequently unclamped and excess flash around the moulds was removed using 1200 grit silicon carbide paper. Once ground the samples were de-moulded, placed in distilled water and incubated in water (37 °C) for 1, 7 and 30 days. Compressive strength, σ_c_, was calculated according to Equation (2) [36]:

(2) $$\sigma_{c}=\frac{4\rho }{\pi d^{2}}$$

where ρ = maximum applied load (N), *d* = diameter of sample (mm).

#### 2.5.2. Biaxial Flexural Strength

Sixty seconds after mixing commenced for each cement, rubber moulds (8 mm Ø, 2 mm thick) were filled to excess with cement and placed between 2 stainless steel plates, clamped, and incubated (37 °C, 1 h). The samples were subsequently de-moulded and incubated in distilled water for 1, 7 and 30 days. The biaxial flexural strength (σ_f_) of the cements was determined in a similar fashion to that of Williams *et al.* [37] which uses three support bearings on the test jig fixed to an Instron Universal Testing Systems (Instron Corp, Norwood, MA, USA) apparatus using a load cell of 1 kN. Testing was performed at a crosshead speed of 1 mm·min^−1^. Five samples for each cement formulation and incubation time were tested. σ_f_ was calculated according to Equation (3) [37].

(3) BFS=ρ(N)t2{0.63In(rt)+1.156}

where ρ = fracture load (N), *t* = sample thickness (mm), *r* = radius of the support diameter (mm).

### 2.6. Adhesive Properties

#### 2.6.1. Sample Preparation

**Figure 1:**
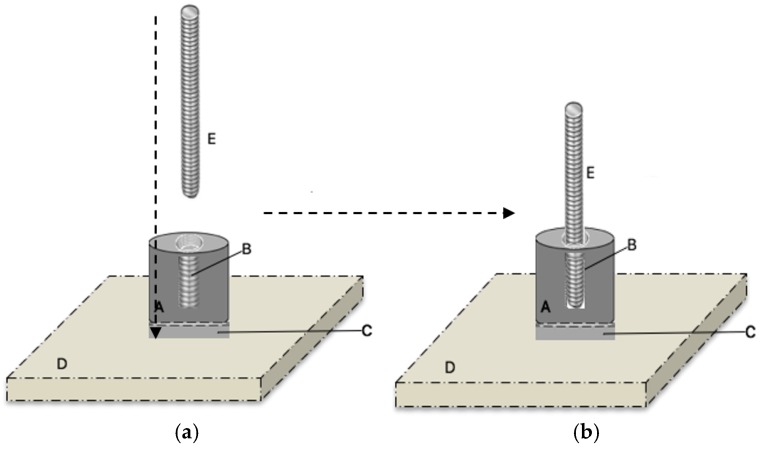
(**A**) displays the Ti Cylinder; (**B**) shows the thread in the center. (**A**) and (**B**) illustrate the materials used in the construction of the test rig; (**C**) displays a layer of one of the GPCs; (**D**) shows the Ti and bone samples used; (**E**) is the anchor that is screwed into the threaded center of the Ti cylinder. (**a**) The Ti cylinder with a layer of cement attached to the center of the Ti and bone samples, then stored in water for 1, 7 and 14 days; (**b**) After storage, the anchor was screwed into the Ti cylinder prior to testing.

To reflect the clinical situation as closely as possible, the adhesive properties of each of the GPCs to a titanium alloy (McMaster Carr, Aurora, OH, USA) were determined using a method comparable to ASTM B348 [38]. Figure 1A,B illustrates the materials used in the construction of the test rig. A layer of the GPC (KBT01, KBT02 and KBT03) was applied to a 19.05 mm diameter cylinder of titanium alloy (McMaster Carr, Aurora, OH, USA), which was then attached to both fresh ovis aries cranial bone and titanium plate (ASTM B265) [39]. Figure 1C displays a layer of one of the GPCs, Figure 1D shows the Ti and bone samples used and Figure 1E is the anchor that is screwed into the threaded center of the Ti cylinder. Five specimens were prepared for each cement composition. Materials were then left to mature (1 h, 37 °C) before being submerged in a single container of distilled water (37 °C). The procedure was repeated for samples submerged for 1, 7, and 14 days.

#### 2.6.2. Adhesive Test

**Figure 2:**
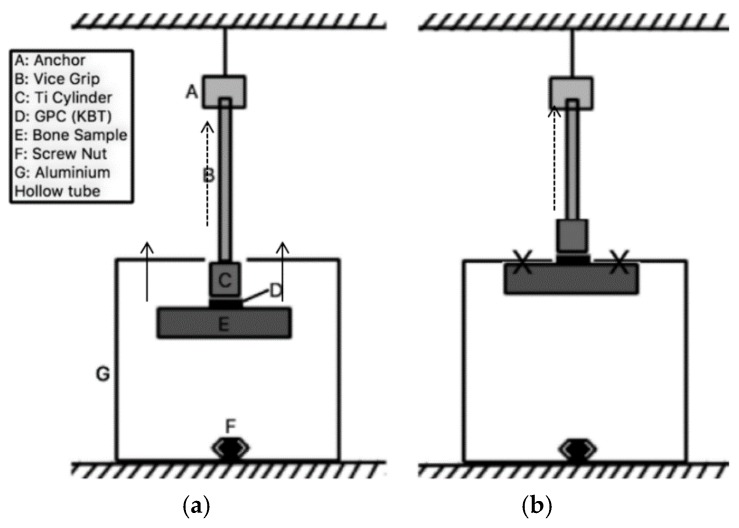
Bond Strength Test. (**a**) The start of the test; (**b**) The bone samples hit equally flat on the X points for both sides, the construct is elevated at 1 mm/ min then the tensile bonding test begins and continues upwards until the failure occurs.

Following maturation in distilled water, the constructs in Figure 1 were individually placed inside a hollow aluminum tube (203 mm × 203 mm with a 25 mm hole drilled through the top; Figure 2) with the Ti cylinder penetrating the hole. The top of the cylinder was then attached to the upper platen of an Instron Universal Testing System (Instron Corp, Norwood, MA, USA) fitted with a ±2 kN load cell. The upper platen was moved upward at a crosshead speed of 1 mm·min^−1^. The test was performed in ambient air (23 ± 1 °C). Samples were tested at *t* = 0 and after 1, 7 and 14 days and converted from Force (N) into tensile strength using Equation (4) below.

(4) σ=FA

where ρ is the bond strength (MPa), *F* is the maximum force applied (N) and *A* is the bonded area (mm^2^).

### 2.7. Ion-Release

The concentrations of Si, Zn, Sr, Na, Ge and Ca ions released from the cements were determined by analyzing the water extracts in which samples of each set cement were stored using a Perkin Elmer atomic absorption spectrometer 800 (AAS800, Perkin Elmer, Waltham, MA, USA). 10 mL aliquots of deionized water were kept at 37 °C in lidded containers. Samples (*n* = 5) of each cement (8 mm Ø, 2 mm thick) were then stored for 1, 7 and 30 days in water. Following removal of cement samples from their aliquots, a 1:10 dilution of the storage water was made using purified water. Calibration standards for Si, Ge, Ca, Zn, Na and Sr elements were prepared from a stock solution on a gravimetric basis. Five target calibration standards were prepared for each ion with 0.1, 0.3, 0.5, 0.7 and 1.0 part per million (ppm) concentrations with distilled water was used as a blank. Samples for Ge, Ca, Zn, Na and Sr ion analysis were diluted in a ratio of 1:10; that is, each 1 mL of concentrated sample was mixed with 10 mL of distilled water while samples for Si analysis were diluted in a ratio of 1:30. A pilot study was conducted to determine the appropriate ratio for dilution of all elements. The optimal working conditions are listed in Table 3.

### 2.8. Micro-CT Analysis

Radiopacity of the cements were determined and compared to a cortical bone standard using a General Electric Healthcare explore Locus SP microCT scanner (Milwaukee, WI, USA). An initial scan was performed, giving an overall X-ray image from which an area of focus was then selected for scanning at full resolution (30 μm). Each of the KBT cement samples were paired with an SP3 calibration standard and placed into a full resolution scan. Images were then reconstructed and the density of the cement samples was measured using affiliated software.

### 2.9. Statistical Analysis

One-way analysis of variance (ANOVA) was used to analyze the data for handling and mechanical properties. A *Post hoc* Bonferroni test was used to compare the relative means and to report the statistically significant differences when *p* < 0.05. Statistical analysis was performed using SPSS software (IBM SPSS statistics 21, IBM Corp., Armonk, NY, USA).

## 3. Results and Discussion

### 3.1. Glass Characterization

#### X-ray Diffraction (XRD) 

**Figure 3:**
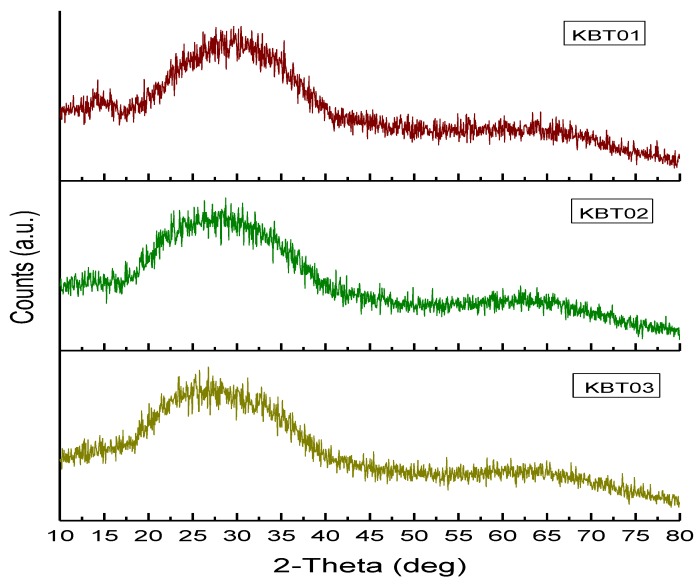
XRD patterns of the formulated glasses (KBT) series.

XRD was performed to determine whether any crystalline phases were present within the KBT glasses (Figure 3). The BT101 glass was sourced from a batch previously confirmed to be amorphous [27].

### 3.2. Particle Size Distribution Analysis (PSA)

All glasses when evaluated by particle size analysis, return a d50 value of between 8.32 μm and 8.69 Particle size of the glass phase will impact both rheology and mechanical properties of GPCs formulated from them; for example, increases in the surface area of the glass component will reduce setting time [40] and likely increase compressive strength [40]. However, the comparable particle sizes between sample sets in the glasses herein would not be responsible for any recorded changes in setting chemistry and so it is fair to conclude that any measurable changes in handling and mechanical properties of the cements being made from these glasses would be related to the chemistry of the glass phase, rather than any differences in physicality.

### 3.3. Scanning Electron Microscopy and Energy Dispersive X-ray Analysis (SEM-EDX)

**Figure 4:**
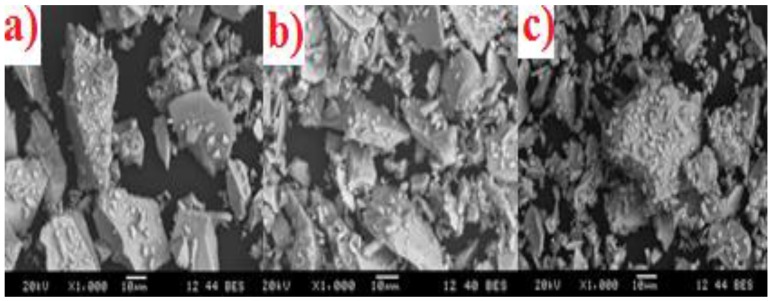
SEM micrographs. (**a**) KBT01; (**b**) KBT02; (**c**) KBT03.

Each glass was examined by SEM (Figure 4a–c). The KBT01 (a), KBT02 (b) and KBT03 (c) glasses were all reported to have a similar particle size distribution (d50), also the SEM imaging show that the particles are not spherical which explains some of the variation in particle size.

EDX was performed during microscopy to confirm that the ion contents incorporated in the starting mixtures for glass firing were present in comparable amounts in the glasses (Table 4) formulated from them.

### 3.4. Calculation of Network Connectivity

Calculation of Network Connectivity for the KBT and BT101 glasses as shown in Table 5.

### 3.5. Cement Handling Characteristics (T_w_ and T_s_)

**Figure 5:**
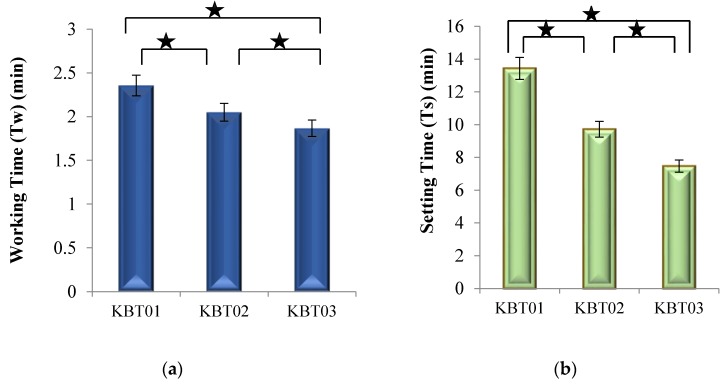
(**a**) Working times of the cement series; (**b**) Net setting times of the cement series. Stars and bars show statistical significance (*p* < 0.05).

Figure 5a displays the working time (*T_w_*) of the cement series formed form the glasses as the concentration of Ge in the KBT glass phase increased from 0.00 to 0.06 mol %.

As Ge addition in the glasses increased, *T_w_* of the cements made from them linearly decreased. Figure 5b displays the setting times (*T_s_*) of the cement series, with *T_s_* decreasing from 800 s for KBT01 cement to 450 s for KBT03 cement. All of these results show a statistically significant difference (*p* < 0.05) with Ge incorporation. The decreasing trends experienced in both *T_w_* and *T_s_* resulted from the introduction of Ge to the glass phase, which may result in an increased susceptibility to acid attack, the glasses releasing more cations into the environment resulting in increased carboxylic (COO-)—metal bonding rates [41]. The introduction of Ge ions, which have a 4^+^ charge, may also increase the bonding rate of the unbonded COO- molecular chains [30]. The network connectivity (NC) data in Table 5 confirm NC increasing with Ge content.

During the cranioplasty surgical procedure, the surgeon requires adequate time to apply the cement before it begins to set. Cranioplasty procedures, which involve drilling and securing a miniplate with screws, as well as adhering the plate to the bone with cement, have been timed at approximately 8 min (480 s) [42]. A minimum T*_w_* for cements in cranioplasty has not been reported in the literature. However, ISO9917 [36] is a standard that defines rheological properties of water-based cements, such as GPCs, for dental applications, and although it does not specify *T_w_* for GPCs, it identifies a range of *T_s_* between 1.5 and 6 min [36]. All cements in this research exhibit T*_w_* greater than 90 s and *T_s_* less than 800 s and are compliant with ISO9917 [36]. It would be beneficial to secure clinical feedback of these cements with the objective of determining the suitability of their rheology for cranioplasty applications.

### 3.6. Compressive (σ_c_) and Flexural (σ_f_) Strengths

σ*_c_* was evaluated according to ISO9917 [36]. Samples were submerged in distilled water for 1, 7 and 30 days prior to testing.

**Figure 6:**
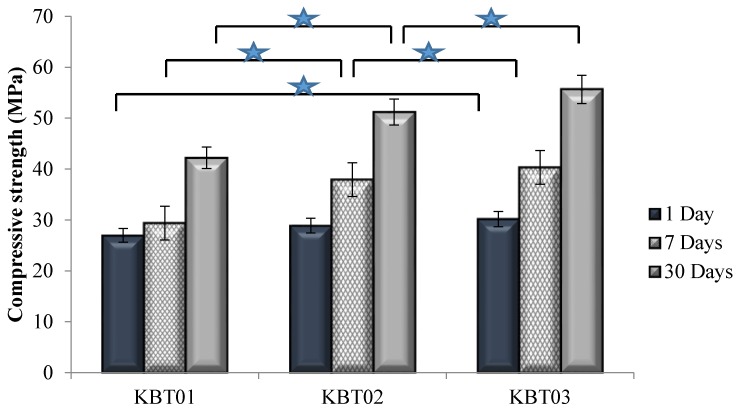
Compressive strengths for the cement series over 1, 7 and 30 days maturation. Stars and bars show statistical significant difference (*p* < 0.05).

Figure 6 displays σ*_c_* recorded for all cement formulations. The compressive strength increased with maturation for each sample set, with the largest increase occurring between 7 and 30 days. The incorporation of Ge in the KBT glass phase also increased compressive strength, with statistical differences occurring between the KBT01 and KBT03 cements at 1 day maturation, and for all cements between 7 and 30 days maturation.

**Figure 7:**
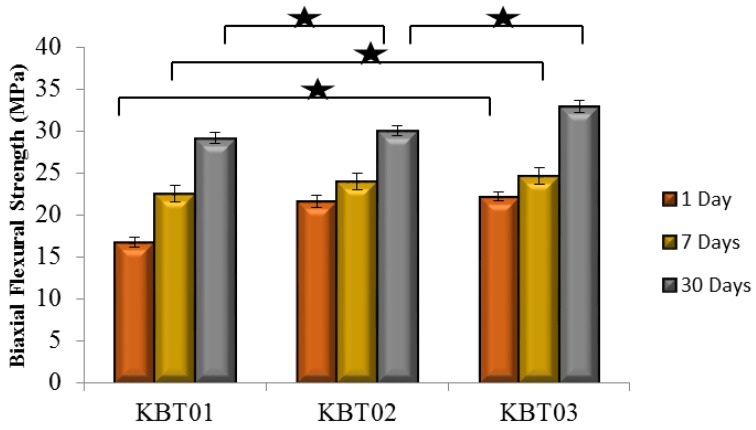
Biaxial flexural strengths for the cement series over 1, 7 and 30 days maturation. Stars and bars show statistical significant difference (*p* < 0.05).

Flexural strength testing was conducted. Figure 7 presents the σ*_f_* results, which exhibited changes in strength with respect to time, with similar trends to σ*_c_* in terms of maturation and Ge addition.

Strength increases with maturation are commonplace with GPCs and are related to the ions released from the glass chelating with the COO^−^ from the acid component over time [43,44,45,46], through a continuous acid-base reaction [47]. In addition to maturation, strengths also increased with Ge content. Ge is a 4^+^ valency ion thus it will bond and increase the network connectivity (NC) of the cement, (Table 5), Hill *et al.* [48] described the entanglement of polyanion chains during GPC setting and how they limit lateral movement, while interactions with neighboring chains limit longitudinal movement. Thus, it is probable that relations between multivalent Ge^4+^ ions, or complexes thereof, interrelate with more than two polyanions to increase chain bonding and entanglement thus creating stronger cements [49,50,51].

### 3.7. Tensile Bonding Test

**Figure 8:**
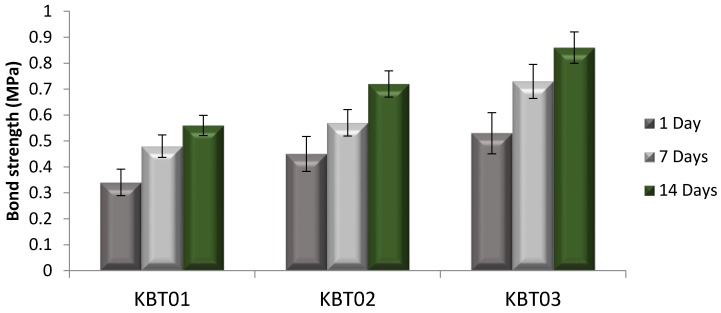
Tensile strength measurements for the bond strength tested over 1, 7, and 14 days.

Figure 8 illustrates the bond strength measurements for the cement series after 0, 1, 7 and 14 days maturation of the bond between the cement samples and bone.

**Figure 9:**
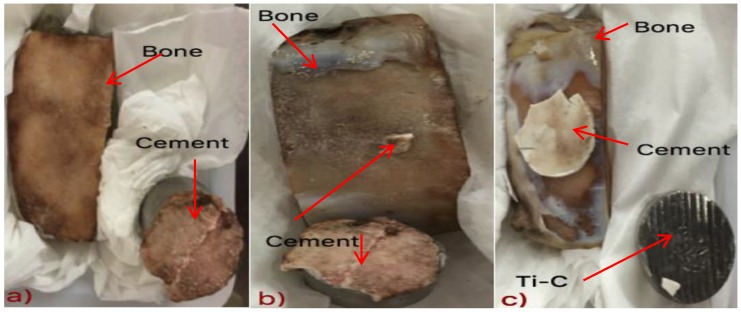
Cement/ovis aries bone constructs after: (**a**) 0 and 1 day maturation; (**b**) 7 days maturation; (**c**) 14 days maturation.

The bond strength between the cement and bone was found to increase with both Ge content in the glass phase and cement maturation (Figure 8). At 0 and 1 day, failure occured between the cement and bone for each construct, yet the titanium cylinder exhibited traces of cement residue (Figure 9a). This cohesive failure was also evident after 7 days maturation, however, as seen in Figure 9b, small parts of the cement remain attached to the bone. After 14 days, failure occured between the Ti cylinder and cement, indicating that the bond strength at the bone/cement interface was greater than that between the Ti cylinder and cement (Figure 9c). Thus, bond strength increased with cement maturation.

Comparing this experimental data to the literature, where five different screws of different lengths were evaluated for securing a titanium miniplate to hydroxyapatite (HA) blocks [5] in a cranioplasty situation, an average bond strength of 97 N, increasing up to 257 N with screw length, were reported [5]. After 14 days maturation, the bond strength using the test methodology described here, averaged between 0.53 and 0.86 MPa (153 N to 247 N), as shown in Figure 9. From this, the KBT03 cement can be considered to offer similar resistance to pull out to the screw method.

### 3.8. Ion Release

**Figure 10:**
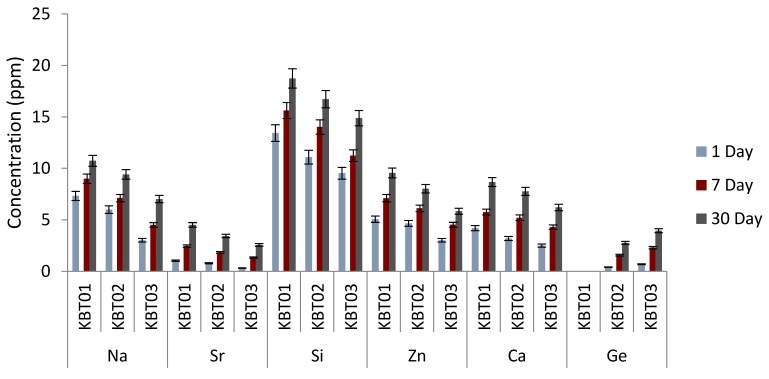
Ion release profiles for the cement series over 1, 7 and 30 days.

Ion release from the cements were measured cumulatively over 1, 7 and 30 days and are tabulated in Figure 10. As would be expected, ion release (Na, Sr, Si, Zn, Ca and Ge) increased with maturation. However, the incorporation of Ge into the glass resulted in lower ion release for the Na, Sr, Si, Zn and Ca ions at the same time point, with the obvious exception of Ge, which, understandably, increases in line with its content in the precursor glass from which it elutes.

Unsurprisingly, the incorporation of increasing amounts of Ge in the glass phase results in increasing amounts of Ge ion release recorded from the resultant cements. There is also less Zn ion release from the cements going through the series as the increased Ge content in the precursor glasses occurs as a result of reduced Zn. However, the quantity of all other ions released from the cements also decreases with increased Ge content, which suggests that Ge is stabilizing the glass network. As the mechanical properties of the cements increase going through the series it suggests that is the Ge ion, with its 4^+^ valency, that is dominating the cement setting reaction.

### 3.9. Radiopacity (X-ray)—MicroCT

**Figure 11:**
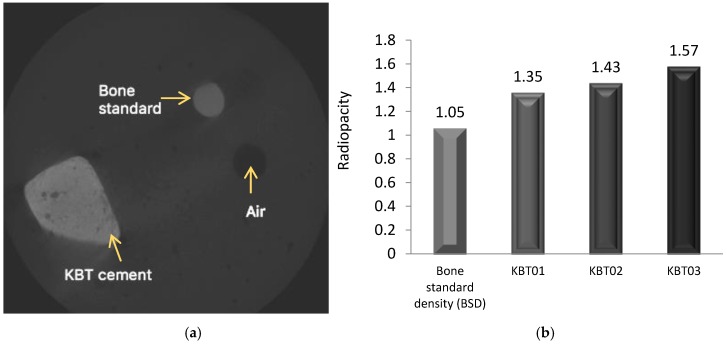
(**a**) Radiograph image of the cement sample and SP3 standard; (**b**) Comparison of the radiopacity of the cements.

Radiopacity was evaluated according to the ASTM F640 “Standard Test Methods for Determining Radiopacity for Medical Use” which is applied for monitoring the position of permanently implanted medical devices [52]. It is vital that orthopedic adhesives are radiologically detectable to facilitate long term monitoring [6]. The results of the radiographic testing are shown in Figure 11(Left). The cement is represented by the lighter colored image compared to the Bone Standard Density (BSD), which is represented as a darker color on the X-ray [53]. As shown in Figure 11(Right), the cement densities were higher (1.35 to 1.57 g/cm^2^) than that of the BSD; the radiopacity of which was measured as 1.05 g/cm^2^.

**Figure 12:**
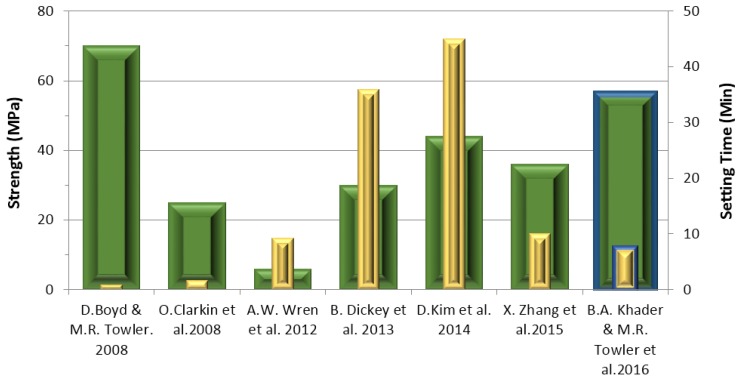
Setting time and compressive strength of aluminum-free GPCs reported in the literature. the blue frame denotes the results of this paper.

Figure 12 describes the setting times and compressive strengths of the Aluminum-free GPCs that have been reported in the literature. In 2008, Boyd and Towler *et al.* [34] developed an ionomer glass, which substituted SrO with CaO; the resultant cements recorded considerably shorter setting times (<1 min) and a maximum compressive strength of 70 MPa. The KBT cement is shown to have a longer setting time (7.5 min) but a slightly lower compressive strength (56 MP) than Boyd and Towler’s study. Wren *et al.* [46] developed an ionomer glass based on the same composition as that of Boyd and Towler but with Ga substituted for ZnO. The setting time of this cement was 9.3 min, similar to that of the cements discussed in this paper. However it recorded a low compressive strength of 6 MPa. The setting time for the KBT glass-based cements was found to be comparable to those recorded by Zhang *et al.* [33] who reported on a cement based on a series of zinc-boron Ge based glasses. However, the compressive strength (36 MPa) reported on Zhang’s cements was lower than that recorded on the materials reported here. Dickey *et al.* [30] also reported on Germanium-glass based GPCs and a further study by Kim *et al.* [54] considered GPCs based on magnesium/strontium-silicate glasses. Dickey *et al.* [30] was found to have had a long-setting time of 36 min while Kim *et al.* [54] had a setting time of 45 min. The strengths and setting times of all of the cements reported in these studies are compared and contrasted in Figure 12.

## 4. Conclusions

The objective of this research was to determine the influence of Ge substitution for Zn on the rheological and mechanical properties of a series of glass polyalkenoate cements (GPCs) derived from novel aluminium-free, ionomer glasses and to subsequently evaluate their suitability for cranioplasty applications by the use of a novel bond test. The handling and mechanical properties of the experimental cements are collated in Table 6.

The KBT03 glass, when mixed with the other components, produced a cement that has handling properties acquiescent with ISO9917 [36] guidelines while all cements of the KBT glass series set within the required time as outlined by ISO5833 [55] for orthopedic cements. Although these standards are designed for evaluating cements for cranioplasty applications, they do offer a window into the suitability of properties of cements for clinical applications. Compressive and flexural strengths of cements were found to be dependent on both the presence and amount of Ge in the glass phase and maturation time. Ion release profiles were also dependent on the presence of Ge in the glass phase, resulting in a smaller amount of Si, Zn, Ca, Na and Sr ions released from KBT02 and KBT03, presumably due to the addition of Ge. MicroCT was able to distinguish radiographically between a bone standard and the cements, radiopacity increasing with the addition of Ge (Figure 11, right). KBT03 appeared to have potential for cranioplasty as the bond strengths recorded were within the range reported for screws applied in the clinical application [5].

## 5. Limitations of the Study

There are a lack of current ISO/ASTM standards for cranioplasty, meaning that it was not possible to target the aims of the research against such standards. Additionally, no biocompatibility data was recorded for these materials to date; this will be addressed in future studies.

## Figures and Tables

**Table 1:** KT Glass compositions (mol %).

Nomenclature	SiO_2_	CaO	ZnO	Na_2_O	GeO_2_
KBT01	0.50	0.10	0.30	0.10	0
KBT02	0.50	0.10	0.27	0.10	0.03
KBT03	0.50	0.10	0.24	0.10	0.06

**Table 2:** BT101 glass composition (mol %) [13,34].

Nomenclature	SiO_2_	CaO	ZnO	SrO
BT101	0.48	0.12	0.36	0.04

**Table 3:** Operating parameters for AAS.

Parameters	Si	Zn	Ca	Na	Sr	Ge
Lamp current (mA)	5	5	10	5	10	10
Wavelength (nm)	251.6	213.9	239.9	330.2	460.7	265.16

**Table 4:** Composition in wt % as verified by EDX.

Composition	KBT01	KBT02	KBT03
O	44.5	48.1	44.1
Si	18.3	17.7	18.2
Ca	2.8	2.5	2.8
Zn	26.5	22.1	22.1
Na	7.9	7.5	7.6
Ge	-	2.1	5.2

**Table 5:** Calculation of Network Connectivity for all glasses. (NF) network former and (NM) network modifier.

Nomenclature	SiO_2_ Backbone (mol %)						
	SiO_2_ (NF)	CaO (NM)	ZnO (NM)	Na_2_O (NM)	SrO (NM)	GeO_2_ (NF)	NC
KBT01	0.50	0.10	0.30	0.10	0.00	0.00	2.0
KBT02	0.50	0.10	0.27	0.10	0.00	0.03	2.2
KBT03	0.50	0.10	0.24	0.10	0.00	0.06	2.4
BT101	0.48	0.12	0.36	0.00	0.04	0.00	1.83

**Table 6:** Summary of the results from the novel cements.

Composition	XRD	*T_W_*/*T_S_* (s)	σ*_c_*_(MPa)_ _(Min-Max)_	σ*_f_*_(MPa)_ _(Min-Max)_	Ti*-Bone (MPa)	Radiopacity (Density g/cm^2^)
	Analysis	*p* < 0.05	*p* < 0.05	*p* < 0.05	(Min-Max)	
KBT01	Amorphous	141/806	27–42	17–29	0.34–0.56	1.35
KBT02	Amorphous	123/583	29–51	21–30	0.45–0.72	1.43
KBT03	Amorphous	112/448	31–56	22–33	0.53–0.86	1.57

Ti* is the Ti cylinder.
